# Errata

**DOI:** 10.36660/abc.20230806

**Published:** 2023-12-14

Arq Bras Cardiol. 2023;120(6):e20220576

No Artigo Original “Construção e Validação do Protocolo EmpoderACO Direcionado a Pacientes em Anticoagulação Oral com Varfarina”, com número de DOI: https:// doi.org/10.36660/abc.20220576, publicado no periódico Arquivos Brasileiros de Cardiologia, Arq Bras Cardiol. 2023;120(6):e20220576, na página 1, corrigir o nome da autora “Rebeca Priscilla de Melo Santos” para “Rebeca Priscila de Melo Santos”.

Arq Bras Cardiol. 2023;120(7):e20230342

No Artigo de Revisão “Os Melhores Artigos de 2022 nos Arquivos Brasileiros de Cardiologia e na Revista Portuguesa de Cardiologia”, com número de DOI: https://doi.org/10.36660/abc.20230342, publicado no periódico Arquivos Brasileiros de Cardiologia, Arq Bras Cardiol. 2023; 120(7):e20230342, considerar o seguinte: Este artigo foi desenvolvido em conjunto pelos Arquivos Brasileiros de Cardiologia e a Revista Portuguesa de Cardiologia, e publicado em conjunto pela Sociedade Brasileira de Cardiologia e Elsevier España S.L.U. Os artigos são idênticos, exceto por pequenas diferenças estilísticas e ortográficas, de acordo com o estilo de cada revista. Qualquer citação pode ser usada ao citar este artigo.

