# Supplementary material for: Errata
Source: Arq Bras Cardiol. 2023 Dec 14;120(12):e20230806. [Article in Portuguese] doi: 10.36660/abc.20230806 (PMC10789376; doi:10.36660/abc.20230806)
Supplement: Translation

# Translation: Erratum

Arq Bras Cardiol. 2023;120(6):e20220576

In the Original Article “Construction and Validation of a Protocol Targeting Patients on Oral Anticoagulation with Warfarin”, with DOI: https://doi.org/10.36660/ abc.20220576, published in the journal Arquivos Brasileiros de Cardiologia, Arq Bras Cardiol. 2023;120(6):e20220576, on page 1, correct the name of the author “Rebeca Priscilla de Melo Santos” to “Rebeca Priscila de Melo Santos”.

Arq Bras Cardiol. 2023;120(7):e20230342

In the Review Article “The Best Articles of 2022 in the Arquivos Brasileiros de Cardiologia and Revista Portuguesa de Cardiologia”, with DOI: https://doi.org/10.36660/abc.20230342, published in the journal Arquivos Brasileiros de Cardiologia, Arq Bras Cardiol. 2023; 120(7):e20230342, consider the following: This paper was jointly developed by Arquivos Brasileiros de Cardiologia, Revista Portuguesa de Cardiologia, and jointly published by Sociedade Brasileira de Cardiologia and Elsevier España S.L.U. The articles are identical except for minor stylistic and spelling differences in keeping with each journal’s style. Either citation can be used when citing this article.
